# An interview with Ewa Czochrowska

**DOI:** 10.1590/2177-6709.23.3.014-023.int

**Published:** 2018

**Authors:** Ewa Czochrowska

**Affiliations:** Graduated in Dentistry at the Dental Faculty in Warsaw, Poland. » Postgraduated in Orthodontics at the Oslo University, Norway. » Professor at the Department of Orthodontics, Warsaw Medical University. » President of the Polish Orthodontic Society. » Former President of the European Orthodontic Society. » Active member of the Angle Society of Europe and the European Board of Orthodontists.

**Figure:**
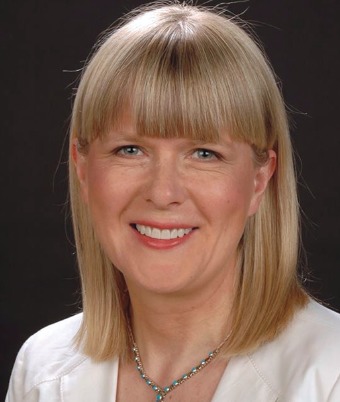

It gives me great joy to interview this great European orthodontist. When I was starting my postgraduate training in Orthodontics in 2002, I got amazed when I read for the first time about autogenic transplantation of teeth in the anterior region. Although this approach is quite common in Europe, it is not a usual treatment option in Brazil. Later on, I got to know the papers published by Dr. Ewa Czochrowska and realized all the scientific background of this treatment protocol. She finished a postgraduate training in Orthodontics in 1997 at the Oslo University, Norway then worked as a Research Fellow at the Orthodontic Department there until 2002. In 2003 she was awarded a PhD from the Oslo University for a thesis on autotransplantation of teeth. For the publication of this work she received the American Journal of Orthodontics and Dentofacial Orthopedics Dewel Orthodontic Award. From 2010 she is working part-time at the Department of Orthodontics, Warsaw Medical University, and has a private orthodontic practice in Warsaw. In 2014 she was awarded a habilitation in medical science from the Warsaw Medical University on her work related to orthodontic treatment of patients with periodontitis and currently maintains a position as associate professor. Her current research is based on the outcome of tooth transplantation, tooth impaction and tooth agenesis and different aspects of interdisciplinary treatment. She has a son who is also a dentist, and her hobbies are trekking in the mountains, arts, jazz and classical music. I hope this interview can therefore bring a new and conservative perspective to treatment plans of very difficult cases we sometimes face in our practices. I must also thank to Dental Press Journal of Orthodontics for the granted honor and privilege to conduct this interview. Now, we may all have a pleasant reading.

É com grande alegria que entrevisto esta grande ortodontista europeia: Ewa Czochrowska. Quando comecei meu curso de pós-graduação em Ortodontia em 2002, fiquei impressionado quando li pela primeira vez sobre o transplante autógeno de dentes na região anterior. Embora esse procedimento seja bastante descrito e praticado na Europa, não é uma opção de tratamento comum no Brasil. Ao longo do tempo, pude ler os artigos publicados pela Dra. Ewa Czochrowska e percebi toda a fundamentação científica desse protocolo de tratamento. A Dra. Ewa terminou seu curso de pós-graduação em Ortodontia na Universidade de Oslo (Noruega), em 1997, e em seguida trabalhou como pesquisadora no Departamento de Ortodontia da Faculdade de Odontologia de Oslo até 2002. Em 2003, ela recebeu seu PhD pela Universidade de Oslo, com uma tese sobre autotransplante de dentes. Pela publicação desse trabalho, recebeu o Dewel Orthodontic Award do American Journal of Orthodontics and Dentofacial Orthopedics. Desde 2010, ela trabalha no Departamento de Ortodontia da Universidade de Medicina de Varsóvia e tem um consultório privado na mesma cidade. Em 2014, recebeu sua certificação em ciências médicas pela Universidade de Medicina de Varsóvia, por seu trabalho relacionado ao tratamento ortodôntico de pacientes com periodontite, onde atualmente mantém cargo de professora associada. Sua linha de pesquisa atual é baseada em transplantes dentários, impacções, agenesias e diferentes aspectos do tratamento interdisciplinar. Ela tem um filho que também é dentista, e seus hobbies são a prática de trekking nas montanhas, artes, jazz e música clássica. Espero que essa entrevista possa, portanto, trazer uma perspectiva nova mas conservadora para o tratamento dos casos muito difíceis que ocasionalmente enfrentamos em nossos consultórios. Também devo agradecer imensamente ao Dental Press Journal of Orthodontics, pela honra concedida para conduzir essa entrevista. Agora, vamos todos aproveitar essa leitura, que acredito ser bastante proveitosa.

Guilherme Thiesen (interview coordinator)

Please briefly describe your current protocol for autotransplants in the anterior region. Guilherme Thiesen

Our protocol for tooth transplantation evaluates first the orthodontic indications and includes treatment planning using cone-beam computed tomography (CBCT), a surgical procedure and follow-up. 

**Figure 1:**
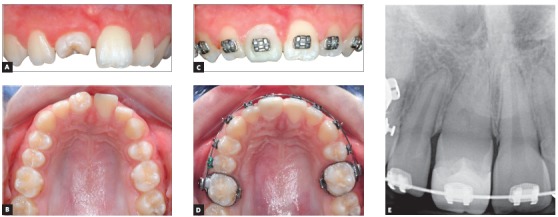
A 9-year-old girl had transplanted a developing upper left second premolar to replace the traumatically lost maxillary right central incisor (A, B). The transplanted premolar was rotated during transplantation to better match the width of the neighboring central incisor. The composite build-up was made 1 year after transplantation and the orthodontic treatment was started to align teeth and establish a normal occlusion (C, D). Normal root development of the transplanted premolar and no hard tissue pathology was present on the intraoral radiograph, except for the pulp obliteration, which is a normal finding in transplanted teeth with developing roots, without a need for endodontic treatment (E).

Agenesis of a lateral incisor is usually treated by canine mesialization or space opening for a dental implant, because a tooth from a posterior region does not match well the morphology of a lateral incisor. For autotransplantations in the anterior region, the indications include traumatic loss of a maxillary central incisor or incisors in children and adolescents (Fig 1). Developing premolars with 1/2 to 3/4 of the root development completed are the best donors, with documented excellent survival and success. Premolar removal should preferably be needed or at least possible from an orthodontic perspective and therefore an orthodontic consultation is always required before performing premolar transplantation. Also, donor selection may be restricted because of root development, however tooth traumas are frequent between 9 and 11 year of age and at this age developing premolars are available. In our team, Dr Paweł Plakwicz, the surgeon, is performing measurements using the CBCT in order to select the premolar which has the best morphology and root development to fit the recipient site. The preferred donors are unerupted premolars with 1/2 of the final root length. Developing premolars can regenerate alveolar bone in patients with traumatic tooth and bone loss, and bone augmentation procedures are not necessary. The surgery starts from a gentle removal of the alveolar bone around the crown of the donor and by checking its mobility, and then the recipient site is prepared with raising the flap and preparing the recipient bed. After the recipient bed is prepared, the donor tooth is gently removed without any injury to its root surface, which may lead to ankylosis and root resorption after surgery, and immediately transferred to the recipient site. If the tooth was unerupted before the surgery, it is placed under the gingival or at the gingival level, resembling the original position within the alveolar bone. The transplant is secured with sutures for 7-10 days and then eruption, pulp healing and root development are observed. Examinations are performed 1 month and every 2-3 months within the first year and then every 1-2 years. Intraoral radiographs and clinical examination of the transplant, to assess its mobility and the status of the neighboring hard and soft periodontal tissues is performed. Pulp obliteration is a normal finding for a transplanted tooth with a developing root and does not require endodontic treatment. Further root development is expected, but sometimes it stops after the surgery, therefore it is advisable to transplant teeth with at least 1/2 of the final root length so the crown-to-root ratio will be satisfactory. If the transplanted tooth does not erupt after the surgery, it may indicate analysis. Early detection of any complications is important, because maybe another donor tooth is still available.

What are the typical orthodontic indications and common complications for premolar transplantation in growing patients? (Ildeu Andrade Jr.)

**Figure 2:**
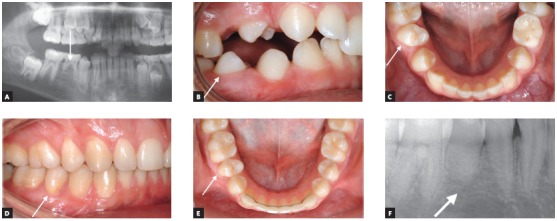
Congenitally missing lower second premolar on the right side was seen during radiographic examination of an 11-year-old boy with Class II division 2 malocclusion and maxillary crowding (A). The roots of the primary second molar at the site of the missing premolar were short. The treatment plan included transplantation of the developing upper right second premolar (arrow) to the position of the congenitally missing mandibular premolar, followed by the fixed appliance treatment to close the extraction space and establish normal overjet and overbite. The transplanted premolar (arrow) erupted spontaneously in the oral cavity six months after transplantation from the subgingival position after surgery (B, C). The fixed appliances were bonded two years after transplantation and eruption of all permanent teeth. Normal tooth contacts were established after orthodontic treatment and overjet and overbite were corrected (D, E). Pulp obliteration and no hard tissue pathology were present at the transplanted premolar (F).

**Figure 3:**
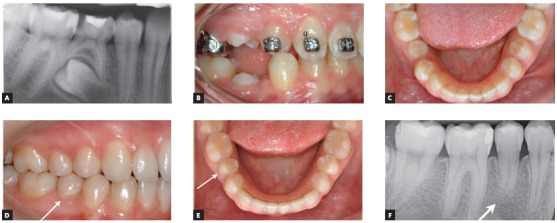
The ectopic position of the developing mandibular right second premolar was present on the panoramic radiograph of a 12-years old girl, who was seeking orthodontic treatment due to malposition of her maxillary teeth and Class II malocclusion (A). The premolar was severely distally tilted and the patient and her parents were interested to perform surgical uprighting (trans-alveolar transplantation) of the affected tooth instead of orthodontic extrusion to shorten the treatment time. The transplanted tooth erupted after few weeks into an oral cavity (B, C) and an orthodontic bracket was bonded after eruption into contacts with the opposing premolars. Normal occlusion was established after orthodontic treatment including the transplanted premolar (D, E; arrow). The root of the transplanted premolar continued development after surgery (arrow), but remained somehow shorter than normal (F). Pulp obliteration and normal hard periodontal tissues are also present on the radiograph.

The most common indications for premolar transplantation in growing patients include congenital agenesis (Fig 2), traumatic injury of maxillary central incisors (Fig 1) and combinations of the above. Also, surgical uprighting (trans-alveolar transplantation) of severely impacted premolars can be considered (Fig 3). Often transplantation of upper premolars is performed in patients with Class II and agenesis of lower second premolars, which is the most frequent tooth agenesis after agenesis of third molars (Fig 2). In those patients, orthodontic space closure is contraindicated in the mandible. Therefore, if the long-term prognosis for preservation of second primary molars is unfavorable, autotransplantation of upper premolars can be considered after careful evaluation of the occlusion and the profile. However, the use of skeletal anchorage to assist orthodontic space closure expands the possibilities of orthodontic tooth movement, but it is easier to close spaces in a maxilla than in a mandible. 

The most common complications after transplantation of premolars with developing roots include pulp necrosis, various types of root resorption including ankylosis and restricted root development. 

What guidelines are given to the child’s parents when recommending autotransplantation in the anterior region? (Eduardo Franzotti Sant’Anna)

I discuss possible alternatives and present strengths and limitations of different treatment options, if available, in order to obtain an informed consent. I am presenting clinical examples of different treatment outcomes, so the patient and his parents can see the final results. Especially the esthetics of the tooth substitution is very important, because patients would not be happy with an aesthetically unacceptable tooth in the smiling zone. Often, they ask me which option I would choose and usually my reply is premolar transplantation. Then I refer the patient to the surgeon, for surgical assessment. He performs the CBCT in order to assess if the morphology of the recipient site allows for accommodation of a specific donor, or donors if multiple teeth are missing. He explains to the patient and his parents the surgical procedures and the follow-up and he is also assessing patient cooperation during this delicate surgical procedure. He performs tooth transplantation in his private office and he feels more comfortable doing the procedure with local anesthesia. General anesthesia is an option and it is up to the operator to decide, but usually it is not necessary - most of the transplantations of developing premolars are performed on 9-13 years old patients. We do not have complaints regarding surgery and post-operative care, but we spend adequate time to explain the procedure and we obtain an informed consent. In a study of Plakwicz et al,^1^ which also assessed the patient’s acceptance of the procedure, the patients responded quite favorably regarding the surgical procedure. However, the decision must be on an individual basis and other treatment options should be considered, especially if the child is psychologically very immature or the discussion with parents is difficult. 

Your success rate for autotransplantation in the anterior region is high. How do you deal with failures? What is the salvage treatment or next step? (Eduardo Franzotti Sant’Anna)

Our success rate is over 90% for developing premolars transplanted to the anterior maxilla, and similar results have been also reported in the orthodontic literature. However, failures can always occur. The failure we most fear is ankylosis of the transplant and it is directly related to the injury of the root surface during surgery and unfortunately it is irreversible. Many times, it can be just spot ankylosis, which involves a minimal part of a root surface. In growing patients ankylosed teeth do not follow the normal growth and development of the alveolar process, thus developing infraposition. Spot ankylosis is practically impossible to detect on radiographs, and the best way to confirm it is by applying orthodontic traction to the suspected transplant. If the transplant does not respond to orthodontic forces, while neighboring teeth are moving, then the ankylosis is confirmed. 

If the transplant becomes ankylosed, the best solution is to extract it and perform another transplantation, if another donor is available. We have had a few cases with ankylosed transplants in which we performed a second transplantation. In those cases, the second transplantation was successful and no ankylosis had developed. Therefore, it is very important to diligently follow transplants, for early failure detection so that other available alternatives can be applied. If there is no available donor, then we can consider extraction of the affected transplant and orthodontic space closure. Therefore, it is advisable to select a donor tooth from another segment or jaw. If orthodontic space closure is contraindicated then we can consider extraction of a transplant and prosthodontic replacements like adhesive FPD. We can also observe the ankylosed transplant since the extent of the infraocclusion is age (younger patients have more growth potential left than older patients) and growth pattern (vertical growers grow more than horizontal growers) dependent.

Could you tell us some specific hints for the surgeon, the orthodontist and/or the future cosmetic procedure when performing autotransplants in the anterior region? (Guilherme Thiesen)

Generally, tooth transplantation is a gentle surgical procedure and it is very important not to cause any iatrogenic damage to the root surface of the donor tooth. Therefore, the easiest type of transplantation is premolar-to-premolar in agenesis cases, when a primary molar is present (Fig 2). Transplantation of a premolar to anterior maxilla can be more demanding in patients with concomitant traumatic loss of alveolar bone and successful accommodation of a donor is difficult (Fig 1). Also impacted teeth or third molars are much more difficult to be removed and the risk of injuring the tooth surface is increased (Fig 3). 

The best candidates for tooth transplantation are patients with a missing tooth at one location of a dental arch and an indication for a tooth extraction at another location. Then, even if the transplantation is unsuccessful there is no negative effect to the occlusion, since tooth extraction was already part of the treatment plan. But often we do not have such clear indications for tooth extraction at the donor site and we must weigh the pros and cons of such a decision, from an orthodontic perspective. Each case must be individually analyzed and discussed with a surgeon.

When premolars are transplanted to the anterior maxilla they must be reshaped to incisor morphology. Enamel reduction should be avoided until the root of the transplanted premolar is fully developed, and this depends on the stage of root development at the time of surgery. I would advise to wait at least 6 months for any reshaping of the transplant, to allow for undisturbed healing. My experience is that young patients, probably until 11 or 12 years of age, do not have problems to have a premolar in the anterior maxilla, because they and their friends are not that concerned about smile esthetics as older patients are. Mothers are often very concerned about this aspect, but I explain to them the importance of successful healing for the long-term success of the transplantation. Initially, the transplanted premolar is reshaped with a composite without enamel grinding. Our study has shown better esthetic results when transplanted teeth after surgery were first orthodontically aligned to allow optimal reshaping.^2^ This should be thoroughly discussed with the patient and the parents before starting the treatment. Porcelain veneers for transplanted teeth have the best esthetic outcomes in terms of color and shape. Contemporary veneers require minimally invasive preparation and thus can be used even in adolescents, but I would wait at least 2-3 years after transplantation of a tooth with a developing root before the final restoration.

What is the oldest patient you successfully autotransplanted in the anterior region? Any specific characteristics of the case that stood out? (Eduardo Franzotti Sant’Anna)

I do not exactly remember the oldest patient in whom we have performed tooth transplantation because we are generally performing transplantations of developing teeth, and complete root development is a limitation of such treatment. Generally, transplantation of developing premolars is performed between 9 and 13 years and transplantation of developing third molars is performed between 15 and 20 years. Recently we have performed few transplantations of mature premolars at ages 15-16 and so far, they are successful, however, this is potentially a less predictable treatment and we do not have enough data to draw a valid conclusion. There are other teams that are performing transplantation of mature teeth and they have published successful cases. 

I do remember the transplanted premolar with the longest observation time after transplantation. This was 41 years after the surgery, at the Department of Orthodontics in Norway, while I was evaluating transplanted teeth 17-41 years after surgery.^3^ The clinical and radiological status of the transplanted premolar was very good and did not differ from natural teeth. I have attained a lot of confidence in transplantation of developing teeth after this study.

Could you compare the effectiveness and efficiency of tooth transplantation and dental implants when replacing missing maxillary incisors? (Ildeu Andrade Jr.)

Dental implants should be applied after growth has ceased, while transplantation of developing premolars can be performed in growing patients, so those two methods have different indications. Transplantation of developing third molars coincides better with the timing of implant insertion, but their morphology is usually much less favorable than premolars, for anterior maxilla transplantations. However, the long-term performance of dental implants is less predictable than dental transplants. Transplanted teeth, if successful, behave like normal teeth with time, and show excellent hard and soft tissue adaptation (Fig 1). They adapt well to age changes and can always be orthodontically moved, if necessary. Implants can develop infraposition even in adult patients and the resorption of the labial plate cannot be excluded, leading to the exposure of labial parts of the fixture, which can compromise the long-time esthetics. Implants do not respond to orthodontic forces and behave as ankylosed teeth due to the osseointegration; this can be an issue for some patients in the long-term. Therefore, there is an increased interest in tooth transplantation as an alternative to dental implants when feasible and indicated. Transplantation of mature teeth can be an alternative to dental implants, but we do not have enough clinical experience with this type of transplantation to reliably compare those two alternatives. 

Based on your clinical experience, it is clear the choice for conservative and essentially biological interventions, including patients with clefts, considering that you have also indicated autotransplantation to these patients. What are the important points to evaluate in these cases, taking into consideration the restrictions/limitations imposed by the presence of the cleft? (Perpétua Freitas)

We have published a case-series on the outcome of premolar transplantation in patients with cleft.^4^ We have assessed 5 transplanted premolars in patients with different types of alveolar clefts and two incisors missing on the cleft side and compared them with a natural premolar in the same patient. The survival and success rate was of 100%, so the results were very encouraging and we have considered premolar transplantation in cleft region of the maxilla as a viable option. In two of those patients, premolar transplantation was performed 1-1.5 years after bone grafting, so this is also possible. Stenvik^5^ has published the results from animal experiments and has recommended postponing tooth transplantation, if planned, at least 4 months after bone grafting. Tooth transplantation into freshly grafted bone can lead to root resorption of the donor. However, it is more difficult to perform premolar transplantation in a deficient maxillary alveolar process, and therefore I would be reluctant to start with tooth transplantation in those patients, since there is a potentially higher risk of failure even for an experienced operator. Also, we know that tooth agenesis is more common in patients with clefts, so availability of a donor might be a problem in those patients. Pawel Plakwicz is collaborating with the Cleft Clinic in Warsaw for the last years for premolar transplantation in patients with clefts. He did few cases and the results are very encouraging not only for survival and success of the donors, but also for the potential regeneration of the alveolar bone in the presence of successful transplants. Transplanted developing teeth during eruption can induce bone formation and preserve continuity of the alveolar process, which is very important for those patients. However, our experience is limited to few cases and these preliminary findings should be interpreted with caution.

What are the great barriers that make the auto-transplants so unusual at a clinical practice (at least here in Brazil and some other parts of the world)? (Guilherme Thiesen)

I think that firstly, it is not taught during undergraduate and postgraduate training, so a lot of the graduates are not familiar with this treatment and its possibilities. Also, patients with tooth agenesis or tooth traumas are usually seen by pedodontist or orthodontists, who traditionally do not perform the surgery. Therefore, interdisciplinary cooperation is necessary and this may be complicated, because also surgeons are not trained in tooth transplantation. Interdisciplinary cooperation is a challenge for busy practitioners and also for patients and their parents. An additional important aspect is that this treatment is performed in children and adolescents, and surgeons are more used to work with adults, who are less demanding regarding the communication during the surgery. I think that dental implants are very widely used and this treatment is very much supported by the companies. Tooth transplantation is not sponsored by any company, because it is a natural replacement, so it can be only popularized by us. In the recent years there is an increased interest in tooth transplantation, especially in the anterior maxilla, because of unpredictable long-term outcome of dental implants. I know that there are some clinicians who are experienced in tooth transplantation in Brazil, because I met them at our congress on tooth transplantation in Sopot, Poland in 2016. Also, some Brazilian doctors came this year in May to Rotterdam for the 2nd Congress on Tooth Transplantation (www.toothtransplantation.com).

In relation to dental impactions, dilacerations represent one of the most challenging etiological factors, since they impose difficulties to forced orthodontic eruption and to tooth accommodation in the arch, especially in the anterior region, due to the limited bone thickness. Could you describe your routine and care in diagnosis and planning in these cases? (Perpétua Freitas)

Dilaceration of the maxillary central incisor is related to a previous trauma in the primary dentition. As a result, the root of the affected incisor is bended in relation to the clinical crown. Treatment options depending on the angulation and direction of the bending may include orthodontic extrusion, surgical extrusion (trans-alveolar transplantation) and extraction. We have performed few trans-alveolar transplantations of dilacerated maxillary central incisors to their normal position. One of those dilacerated incisors was rotated 180 degrees to accommodate the root, which was bend towards the labial part of the alveolar process. But the prognosis for trans-alveolar transplanted central incisors is much more uncertain, due to much more difficult surgical access and tooth morphology. Then injury to root surface is much more likely, often leading to the extraction of a severely dilacerated maxillary central incisor. In those cases, it is worthy to consider mesialization of a neighboring lateral incisor and subsequently of all teeth in that segment. Feldspathic porcelain veneers are the best choice to reshape the mesialized lateral incisor and often the canine. The advantages of lateral incisor mesialization to the position of the extracted central incisor over a dental implant include the excellent long-term hard and soft tissue adaptation and the possibility to perform it in children and adolescents who had limited possibilities for tooth replacement. 

Could you tell us a little bit about the Orthodontics specialty in your country (Polland) and in Europe? How many Orthodontic Programs are there in Polland and Europe? How are they evaluated and regulated? (Ildeu Andrade Jr.)

General guidelines for postgraduate training in Orthodontics in Europe and in Poland follow the Erasmus guidelines established in 1992 and implemented in all postgraduate orthodontic programs in the European Union (Poland is a part of European Union). This is minimum 3-years postgraduate training with a described in details specific theoretical and practical clinical part. It ends with a final theoretical and practical examination, which usually includes presentation of treated cases. The curriculum also usually requires for the students to conduct some sort of research, which can also be presented in the form of a review article. It can vary from country to country, but the basic principles remain the same. The Erasmus guidelines were updated four years ago and the report was published.^6^ Recently, a Network of Erasmus Based European Orthodontic Programs (NEBEOP) was introduced and many orthodontic programs in Europe have joined it.

Postgraduate training in Orthodontics in Poland is Erasmus-based 3 years program. The selection of candidates in Poland is based on the results from a final, national examination after a successful completion of university-based dental education and one-year practical training. The competition is intense and only those with the top grades can be accepted for postgraduate training at designated and government approved centers in hospital and universities. The selection and allocation of candidates is performed on a national basis, which has its strengths and limitations. The final examination includes a theoretical part in a multiple-choice format and practical part with presentation of eight treated cases including five defined categories and the discussion about cases. The theoretical part is very demanding and only about 50-70% are passing. It can be repeated every six months. To my knowledge, every year between 10 to 20 candidates become specialists in Orthodontics, who are registered at the national registry for specialists in dentistry.

Being a teacher is a challenge. What aspects of the profession inspire you and give you the most pleasure? What aspects would you like to change? (Eduardo Franzotti Sant’Anna)

I think that technological progress is important, but it must be seen in the light of professional excellence and really to add to what we have. This is something I would like to teach the students and also show them what can be achieved with traditional orthodontic treatment. However, I believe that the most important is to teach them that their treatment must be for patients’ benefit in short term and also in long term. We must listen to our patients and we must provide therapies that respect their needs. But of course, patients do not know what is possible and what is important, and we must have adequate knowledge and long-term perspective to advise them about their future needs. Orthodontics is an elective treatment, since no one dies because of malocclusion; but at the same time, we can significantly improve the smile and facial esthetics and often function. The ultimate goal of our treatment is a satisfied patient, but satisfaction is related to pre-treatment expectations and the experience during the treatment. Thus, it is very important to discuss with a patient what is expected, what can we achieve and how it will be maintained. This is often neglected by the students, who are focused on details on how to manage orthodontic devices or are excited to try new things. 

I would like for us to be good medical providers and deliver a treatment that addresses the patients’ needs and is effective and reliable in long-term. We cannot achieve it without considering the scientific evidence. Clinicians usually have limited knowledge on how to read and assess scientific reports and in planning reliable clinical trials. This is what I wish to change, because then we can listen more critically to the commercial claims and make valid clinical choices based on the evidence, and not unsubstantiated promises. Companies are needed to provide us with tools to treat our patients, but sometimes they are too aggressive and we must interpret their claims with a caution. We should always perform evidence-based medicine with an aim for a clinical excellence. Tooth transplantation is using biology to make the treatment effective, but still reliable studies are needed to guide our clinical decisions.

You have traveled the world and have met many orthodontists. Who are the two who have influenced you the most and why have they done so? (Ildeu Andrade Jr.)

Undoubtedly, Arild Stenvik and Bjorn Zachrisson, who were my professors in Oslo when I was a postgraduate student and who have supervised my doctoral thesis at the Oslo University. They were excellent clinicians and researchers and I feel privileged that they have shared their knowledge with me. Bjorn Zachrisson is a world-known lecturer and I could learn from him how to master clinical presentations. Why did they have chosen to guide me? I really do not know! Perhaps, they saw in me that I was truly interested in Orthodontics and in tooth transplantation, and that I was willing to work hard to both learn and satisfy their expectations. I feel fortunate, but I hope that they are also happy with my progress in tooth transplantation. I would like to mention one more orthodontist, who had inspired me to become an orthodontist 27 years ago. It is Jonathan Sandler from UK, who guided me into the world of contemporary Orthodontics, because I have obtained my dental training in communist Poland, where only removable orthodontic appliances were available at the time. Jonathan has followed my professional development closely over the years and he was the one who introduced me to the Angle Society of Europe. But there were so many great clinicians and researchers who I met in my life and to whom I am so grateful that they have shared their knowledge and passion for excellence in Orthodontics. 

Please tell me about your ongoing research. Is there a particular project you are most excited about? (Eduardo Franzotti Sant’Anna)

I have seven doctoral students at the Oslo Medical University with various projects related to impacted teeth, tooth agenesis, dental implants, extraction of teeth in Orthodontics and TMJ. My habilitation thesis was related to orthodontic treatment in patients with severe periodontitis and I am currently preparing the manuscripts for my habilitation. I think that periodontology is an exciting field with a lot of ongoing high-quality research. And since we live longer and caries is well controlled, we can preserve our teeth longer. Therefore, in the future we will have to treat an increasingly aging population, with potential periodontal problems. 

Of course, tooth transplantation is my passion and we have a lot of clinical material to evaluate and publish. My dream is to establish a trauma clinic, which could help the growing patients with traumatic injuries and serve as a consultation and training center for dental students and other specialists in dentistry. It would be a multidisciplinary center with specialists from pediatric dentistry, oral surgery, Orthodontics, endodontics and prosthodontists. I believe that it is very much needed in Poland, since we do not have such designated centers for tooth traumatology. A trauma clinic would also give a possibility for a standardized registration and evaluation of the collected clinical material, including tooth transplantation, which is important in evaluating clinical decisions. Everyone deserves a long-lasting, beautiful smile and I should share my knowledge in Orthodontics and in tooth traumatology for the benefit of our patients. 
